# Membrane Morphology Is Actively Transformed by Covalent Binding of the Protein Atg8 to PE-Lipids

**DOI:** 10.1371/journal.pone.0115357

**Published:** 2014-12-18

**Authors:** Roland L. Knorr, Hitoshi Nakatogawa, Yoshinori Ohsumi, Reinhard Lipowsky, Tobias Baumgart, Rumiana Dimova

**Affiliations:** 1 Department of Theory and Bio-Systems, Max Planck Institute of Colloids and Interfaces, Potsdam, Germany; 2 Frontier Research Center, Tokyo Institute of Technology, Yokohama, Japan; 3 Department of Chemistry, University of Pennsylvania, Philadelphia, Pennsylvania, United States of America; National Institute of Biological Sciences, Beijing, China

## Abstract

Autophagy is a cellular degradation pathway involving the shape transformation of lipid bilayers. During the onset of autophagy, the water-soluble protein Atg8 binds covalently to phosphatdylethanolamines (PEs) in the membrane in an ubiquitin-like reaction coupled to ATP hydrolysis. We reconstituted the Atg8 conjugation system in giant and nm-sized vesicles with a minimal set of enzymes and observed that formation of Atg8-PE on giant vesicles can cause substantial tubulation of membranes even in the absence of Atg12-Atg5-Atg16. Our findings show that ubiquitin-like processes can actively change properties of lipid membranes and that membrane crowding by proteins can be dynamically regulated in cells. Furthermore we provide evidence for curvature sorting of Atg8-PE. Curvature generation and sorting are directly linked to organelle shapes and, thus, to biological function. Our results suggest that a positive feedback exists between the ubiquitin-like reaction and the membrane curvature, which is important for dynamic shape changes of cell membranes, such as those involved in the formation of autophagosomes.

## Introduction

**Figure 1:**
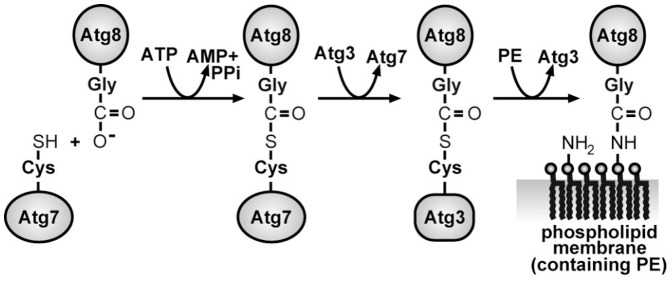
Minimal system to covalently bind the protein Atg8 via an ubiquitin-like reaction to phosphatidylethanolamine (PE).

Even though covalent binding of proteins to phospholipid membranes is ubiquitous in nature, systematic experimental studies investigating this process are quite rare. In the budding yeast *Saccharomyces cerevisiae*, the autophagy-related (Atg) protein Atg8 is the key protein involved in autophagy, a central proteolytic pathway in eukaryotic cells which is involved in several physiological processes as well as in the pathogenesis of many diseases [1]–[3]. As an ubiquitin-like protein, Atg8 binds covalently to an amino group of the target molecule in a complex reaction cascade by (i) cleavage of the carboxy-terminal arginine of Atg8 by Atg4 to expose a glycine residue, (ii) formation of a thioester bond with the E1-like enzyme Atg7 under consumption of ATP, (iii) exchange of Atg7 by the specific E2-like enzyme Atg3, and (iv) conjugation to the substrate, see Fig. 1. The last step can be enhanced by the presence of the E3-like enzyme Atg12-Atg5 or its complex with Atg16 (the Atg16 complex). In contrast to all other ubiquitin-like reactions, the substrate to which Atg8 binds is not a protein but the hydrophilic head of the lipid phosphatidylethanolamine (PE). Thereby, the water-soluble Atg8 becomes anchored as Atg8-PE in membranes. However, the role of this protein and its covalent anchoring is poorly understood.

Strongly curved membranes such as small intracellular vesicles and tubular structures are proposed to be precursors of autophagosomal membranes *in vivo.* [4]–[6] These preautophagosomal membranes form a flat double-membrane sheet, which increases in size, bends and closes into a spherical double-membrane organelle. During these complex morphological shape transformations the curvatures of the membrane as well as the density of Atg8 on autophagosomal membranes change significantly. Theoretical considerations suggested that Atg8 conjugation to PE may change the preferred curvature of membranes [7] thus explaining the size regulation of autophagosomes by Atg8 observed *in vivo* in *Saccharomyces cerevisiae* [7], [8]. However, experimental studies addressing the relationship between Atg8-PE and membrane curvature are not available.

The conjugation reaction of Atg8 to PE has been reconstituted *in vitro* with purified proteins and nm-sized vesicles [9]–[11]. The reaction performed best at concentrations of 60–70 mol% dioleoyl-PE (DOPE) in the target membrane or by inclusion of the second ubiquitin-like conjugation system (the Atg16-complex). This complex is formed after Atg12 conjugation to Atg5 by Atg7 (an E1-like protein) and Atg10 (an E2-like protein) and assembling of two Atg12-Atg5 conjugates with two Atg16 molecules. The Atg16-complex is essential for the reconstitution of Atg8 in cell-sized giant unilamellar vesicles (GUVs) [12], [13]. However, the Atg16 complex can bind directly to lipid membranes [12] and may form a protein scaffold on the membranes together with Atg8-PE, which in consequence decreases Atg8-PE mobility [13]. Thus, the experimental setups reported so far did not allow for a quantitative and well defined reconstitution of Atg8-PE in GUVs, and the presence of the Atg16 complex prevented studying the interplay of Atg8 conjugation, membrane curvature and Atg8-PE sorting.

In this study we developed an alternative system to reconstitute Atg8 in GUVs. The approach requires only the minimal components of the ubiquitin-like reaction excluding the Atg16-complex. We found a complex interplay between the Atg8 conjugation reaction, Atg8-PE localization and membrane curvature.

## Results and Discussion

### Atg8-PE reconstitution in GUVs via minimal conjugation reaction

In a first set of experiments we screened membrane compositions with PE fractions optimized for conjugating Atg8 for their ability to form stable GUVs, for details see Methods. To observe the formation of Atg8-PE on GUVs microscopically, the vesicles were incubated with Atg8 (tagged with green fluorescent Alexa488) and the purified components of the conjugation system Atg3 and Atg7. In all experiments, the membranes were labeled with 0.5 mol% TexasRed-DHPE (TR). Here and below, integrated fluorescent intensities are given in the format 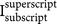, where the subscript refers to the detected molecule (TR or Atg8) and the superscript refers to the origin of the signal – either the free-standing GUV membrane (GUV), the adhesion zone (AZ), the membrane tube (tube) or nm-sized liposomes (lipo). The relative protein density D^superscript^ at the membrane surface is defined as the intensity ratio between Atg8 and membrane dye, D = I_Atg8_/I_TR_.

**Figure 2:**
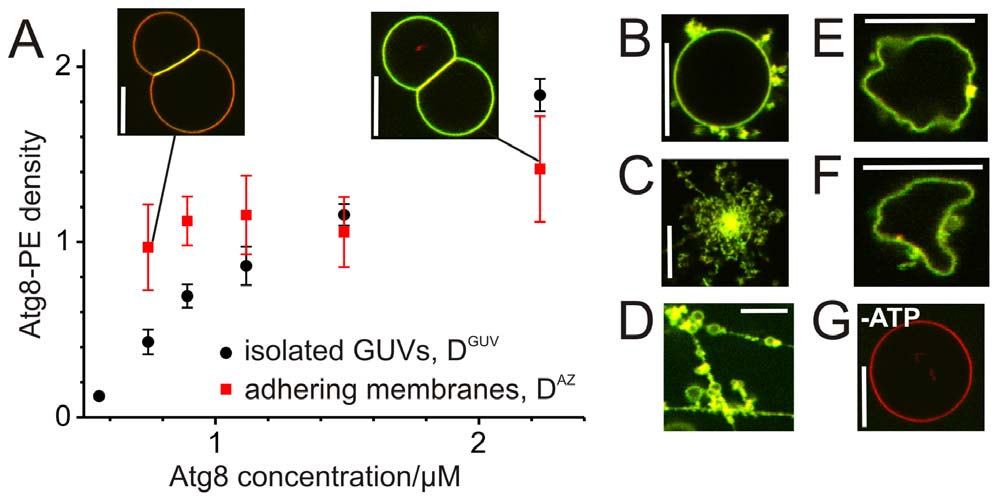
Atg8-PE density on GUVs depends on Atg8 concentration. (**A**) Atg8-PE densities on isolated GUVs (D^GUV^) and in adhesion zones between two GUVs (D^AZ^) as a function of the bulk Atg8 concentration; insets show adhering GUVs for the indicated concentrations; mean ± SEM, n = 9–12 GUVs per concentration. At the highest Atg8-PE density examined, GUVs spontaneously form external protrusions (**B**), while some GUVs disintegrate into tubular networks (**C, D**) or show pronounced non-fluctuating deformations (**E, F**). This behavior is not observed in the absence of ATP (**G**). All snapshots are merged dual color images; scale bars 10 µm.

In agreement with previous data on nm-sized vesicles [9], we detected Atg8 localization to the membranes of GUVs with high content of PE, Fig. 2. Such localization was not found in the absence of ATP, which indicated successful conjugation of Atg8 to PE, Fig. 2. In contrast to previous work [12], the distribution of Atg8-PE on the surface of single vesicles was uniform. The variation of Atg8-PE density within a GUV population was small for the whole range of protein concentrations explored. This allowed us to perform quantitative measurements of Atg8-PE conjugation and we found that the relative Atg8-PE density D^GUV^ depends linearly on the bulk concentration of the protein, see Fig. 2A. We attempted to evaluate the absolute Atg8-PE surface densities employing a calibration procedure based on the use of small unilamellar vesicles [14], [15]. However, our attempts were hampered by Atg8-PE-induced aggregation of the small vesicles. Applying the range of published calibration factors [14] for our sorting experiments, for the surface density we find one Atg8 molecule per 250–3500 nm^2^. This result agrees reasonably well with the roughly estimated density of Atg8-PE *in vivo* (one molecule Atg8 per 2000 nm^2^) [16].

### Membrane adhesion induced by Atg8-PE

Previous studies reported adhesion (tethering) of vesicles induced by Atg8-PE [12], [17], [18]. We observed adhesion of GUVs as well. Interestingly, the Atg8-PE density in the adhesion zones (D^AZ^) between GUV couples varied very little with the Atg8-PE density on GUVs (D^GUV^). As a consequence, a significant enrichment of Atg8-PE in the adhesion zones was detected in the low-concentration regime (Atg8 concentration during incubation below 0.75 µM), whereas the high-concentration regime (above 1.5 µM Atg8) is characterized by a depletion of Atg8-PE in the adhesion zone compared to the free-standing GUV membrane. This effect is demonstrated in Fig. 2A. The insets show adhering GUVs for the low- and high-concentration regimes where both channels are merged.

However for the lowest concentration examined (0.56 µM Atg8) we did not find adhesion of GUVs. This suggests that membrane adhesion induced by Atg8-PE occurs above a minimal conjugate density threshold only. Such a minimal density was suggested theoretically [19] and implies that for the adhesion of Atg8-PE bearing membranes in cells, a minimal number of Atg8-PE is required as well.

Membrane hemifusion activity was reported for Atg8-PE [17], [18] which later was suggested to be caused by high DOPE concentrations [17]. In the case of hemifusion, the adhesion patches between GUVs are expected to consist of only one bilayer assembled from the inner monolayers of both GUVs and, thus, do contain no Atg8-PE. In addition, the intensity ratio of the membrane dye between the adhesion zone and the unbound membrane, 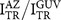, should be equal to one [20]. In contrast, our data implied 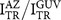  = 1.95 and we detected Atg8-PE within the adhesion zones. These observations suggest that two intact GUV membranes were present in the adhesion zone and they do not exhibit hemifusion.

### Membrane tubulation by Atg8-conjugation

At intermediate Atg8-PE densities, we observed that GUVs tend to spontaneously form external protrusions during the conjugation reaction, see Fig. 2B. Extending the protrusions by micromanipulation showed that they represented collapsed membrane nanotubes. At high Atg8-PE densities, GUV-tubulation became more pronounced and such GUVs were generally fragile and tended to collapse when mechanical stress was applied. They appeared to be tense. In contrast, GUVs fluctuated visibly in the absence (or at low densities) of Atg8-PE. Tube formation consumes membrane area. Thus, membrane tension builds up [21], and vesicle rupture can be expected as a result of the rising tension. In some cases, the spontaneous collapse of GUVs into tubular-vesicular networks was observed, Fig. 2C, D. The tubulation indicates that the Atg8 conjugation reaction can generate membrane curvature. Coupled to ATP hydrolysis, the composition of the membrane is actively altered and the preferred or spontaneous curvature of the membrane is changed. The membrane bends to form curved structures such as nanotubes. Note, that this mechanism is different from simple protein adsorption as previously considered in ref [22]. and that Atg8-PE does not desorb from the membrane.

In agreement with our findings, Atg8-PE was detected on tubular membrane structures in living cells [6] and on GUVs [13]. In Ref. [13], Kaufmann et al. established the reconstitution of Atg8-PE in GUVs with an approach complementary to ours. The approach employed membranes with lower PE fraction and the Atg8 conjugation reaction occurred in the presence of components of the Atg16-complex. Membrane protrusions are visible on all GUV shapshots shown, some GUVs showed extensive membrane deformation, including tubulation and vesiculation. The study suggested that together with components of the Atg16-complex, Atg8-PE forms a scaffold-like protein coat on the surface of membranes. [13] Here, we observed similar membrane deformations at high Atg8-PE densities in the absence of the components of the Atg16 complex, Fig. 2B–F. Our results suggest that (i) the data by Kaufmann et al. are obtained from GUVs with rather high densities of Atg8-PE; (ii) membrane deformations by Atg8-PE occur independently of the membrane composition; and (iii) Atg8-PE alone can trigger shape changes in membranes.

Interestingly, another study reconstituting Atg8-PE on GUVs did not report GUV tabulation [12]. However, the experiments explored conditions very different from ours, e.g. Atg8 was modified with eGFP. The bulky eGFP domain (27 kD) might influence the behavior of the much smaller Atg8 protein (13 kD). Atg8-PE was detected only on a minor fraction of the GUVs and a strong accumulation of Atg8-PE in the adhesion zones was observed. The latter suggests that the Atg8-PE densities attained in ref. [12] correspond to our low concentration regime in Fig. 2A, left inset, where no tubulation is expected.

### Stabilization of high membrane curvature by Atg8-PE

**Figure 3:**
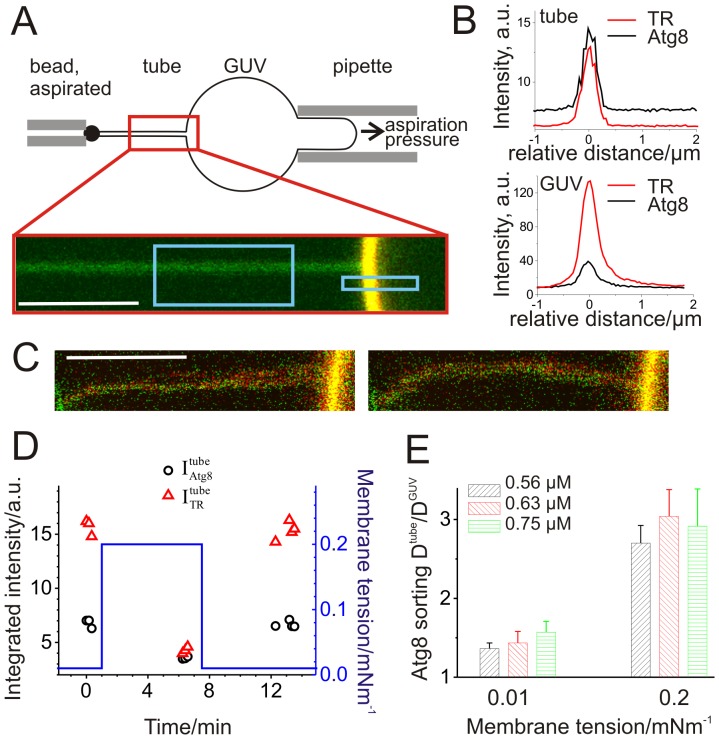
Atg8-PE stabilizes membrane curvature and sorts into strongly curved membanes. (**A**) Setup used for tube pulling. After aspiration, the vesicle containing biotinolyted lipids and the streptavidin coated bead were brought in contact and adhere. Once bound, a tube was pulled by moving the pipette holding the bead, a confocal xy-scan illustrates such a membrane tube pulled from a GUV. (**B**) Fluorescence intensity profiles of the membrane tube (top) and the corresponding GUV membrane (bottom) of the boxed regions in (**A**). (**C**) At high Atg8-PE densities, pulled tubes appeared blurry and exhibit pronounced fluctuations. Two consecutive xy-scans of the same tube illustrate movements of the tube; see also S1 Video. (**D**) Increasing the membrane tension of the GUV at time t = 1 min (blue line, right axis) decreases the radius of the membrane tube from 70 nm to 16 nm and accordingly changes the intensities of the membrane tube and Atg8-PE (data points, left axis). This change is reversible as shown with decreasing the tension at time t = 7 min. For experimental details see methods. Data points correspond to consecutive scans of one individual tube. (**E**) The normalized Atg8-PE densities in the tube D^tube^/D^GUV^ increase with tension. Three different Atg8 concentrations are shown, n = 7 GUVs for 0.56 and 0.63 µM Atg8, n = 3 GUVs for 0.75 µM Atg8, mean ± sem. The scale bars in (**A**) and (**C**) are 5 µm.

*In vivo*, Atg-PE is conjugated to phagophores, sheet-like organelles which consist of a strongly curved rim and essentially flat membrane segments. We speculated that Atg8-PE might distribute differently on membranes with varying curvature and examined the distribution of Atg8-PE in a membrane mimicking such morphology. By micromanipulation of single GUVs, we created one strongly curved membrane tube which is connected to an essentially flat GUV membrane [23], [24] as sketched in Fig. 3A. By changing the aspiration pressure in the pipette holding the GUV, the radius of the membrane tube and, thus, its curvature can be adjusted, see methods. A typical confocal image of the part of the GUV, where the tube is attached, is shown in Fig. 3A, and the corresponding intensity profiles in Fig. 3B. Pure lipid tubes, or tubes pulled from GUVs with low Atg8-PE density appear always straight. In contrast, tubes extracted from vesicles with intermediate or high Atg8-PE density tend to exhibit shape fluctuations away from the linear tubular shape as observed under the microscope, Fig. 3C, even at membrane tensions as high as 0.2 mN/m. A movie of a fluctuating tube is provided as S1 Video. These fluctuations indicate stabilization of membrane tubes mediated by Atg8-PE, in accordance with the observations of tubulation of GUVs containing Atg8-PE, Fig. 2B, and formation of stable tubular networks from disintegrated GUVs, Fig. 2C. Similar stabilization of membrane nanotubes upon protein absorption at high densities was reported recently for absorption of amphiphysin on membranes [15].

### Curvature sorting of Atg8-PE

Increasing the aspiration pressure of GUVs (blue line in Fig. 3D) leads to a decrease of the radius and the membrane area of the tube. Accordingly, the intensity of the tube 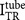 decreased (this process being reversible). The reduction of 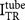 was similar to that observed in protein-free GUVs under identical conditions. The fluorescence signal from the protein, 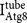, dropped as well, but this decrease was significantly smaller compared to the drop in TR-intensity, Fig. 3D. This can be seen more clearly in Fig. 3E, where tube Atg8-PE densities were normalized by the Atg8-PE densities of the particular GUV, and values larger than one denote sorting of Atg8-PE into tubes. We found that the tubes with smaller diameters (corresponding to higher membrane tension) are enriched in Atg8-PE by a factor of two. Comparing this Atg8-PE curvature sorting for three different Atg8 concentrations, we did not observe any significant influence in the range of concentrations tested, Fig. 3E. Curvature sorting of Atg8-PE located on sheet-like membranes such as growing autophagosomes will result in protein densities varying with the local curvature of the phagophore and as a consequence, induce inhomogeneous spontaneous curvatures on the organelle. Such differences are capable to increase the stability of the open, flat form of phagophores far more than a homogeneous increase of the spontaneous curvature [7]. Thus, curvature sorting amplifies the active role of Atg8-PE in shaping the autophagosomal membrane.

Membrane curvature has been previously proposed as a general factor driving the redistribution of membrane-bound proteins [25]. This proposal was based on data for amphiphilic proteins inserting passively into membranes of nm-sized liposomes displaying different curvatures (single liposome curvature or SLiC assay). As a negative control the water-soluble streptavidin binding to biotinylated head groups of membrane lipids was considered [25]. Similarly to streptavidin, Atg8 is a non-amphiphilic water-soluble protein but it attaches covalently to the head groups of PEs. In contrast to streptavidin, our data demonstrate curvature sorting of Atg8-PE. Thus, our results suggest that the Atg8 conjugation reaction represents not only a simple attachment of a soluble protein to membrane lipids, but also that a direct interaction of the protein domain of Atg8-PE with the membrane exists. The latter might be related to conformational changes of Atg8 observed during lipidation [9].

### Curvature preference of the minimal conjugation reaction

**Figure 4:**
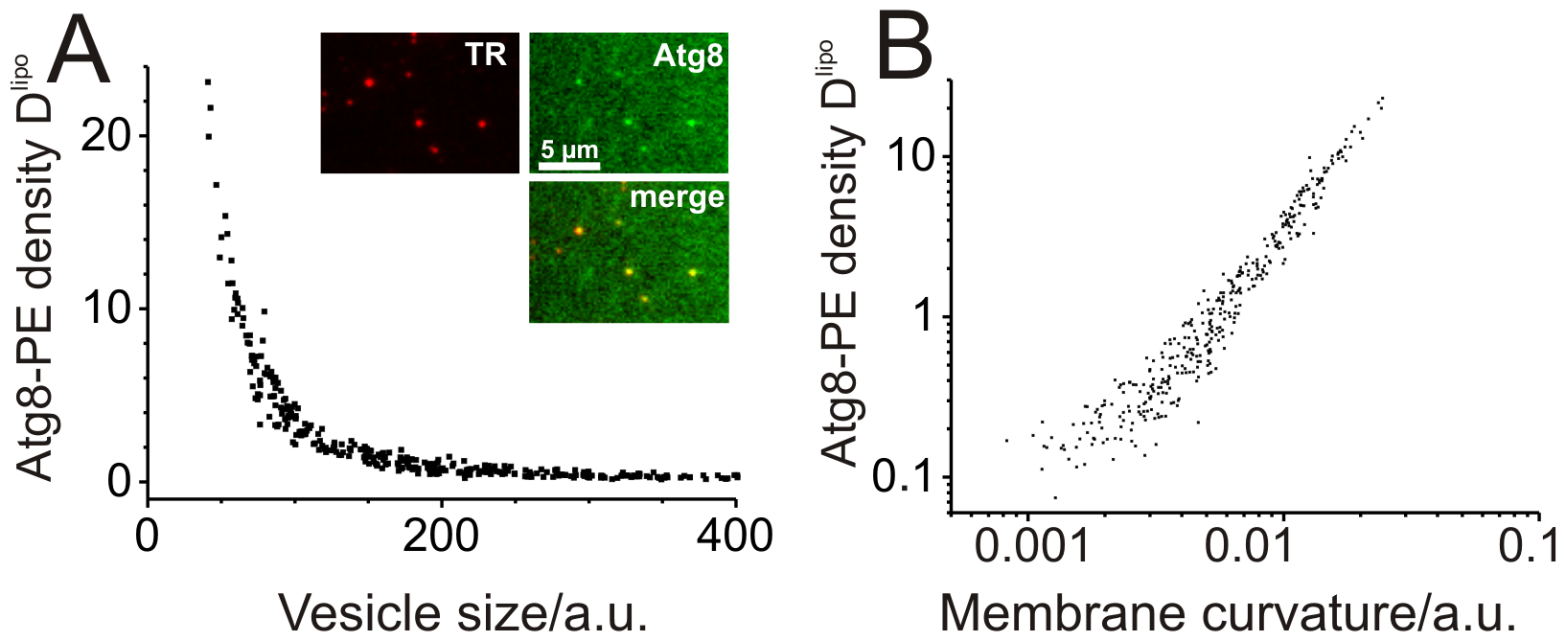
Curvature preference of the Atg8 conjugation reaction. (**A**) Conjugation of Atg8 to individual liposomes of varying size as measured by the SLiC-assay [25]. The Atg8-PE density, 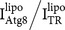, is plotted as a function of the vesicle radius, 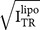, after 20 min incubation with 125 nM Atg8 for n = 366 vesicles. The insets show snapshots of immobilized liposomes. (**B**) Atg8-PE density (same data) as a function of membrane curvature, 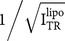.

Strongly curved membranes such as small intracellular vesicles and tubular structures are proposed to be precursors of autophagosomal membranes in vivo [4]–[6]. To address the effect of membrane curvature on the Atg8 conjugation reaction we applied the SLiC assay. Small liposomes, spanning more than a factor of ten in vesicle size, or membrane curvature, Fig. 4, were immobilized on cover slips and the Atg8 conjugation reaction was performed. We measured the integrated fluorescence intensities per liposome, 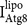 and 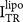, of several hundred individual liposomes. Plotting the Atg8-PE density versus the liposome size, we found that Atg8-PE densities increase with decreasing liposome size, Fig. 4A. In Fig. 4B, the data obtained after 20 min incubation are plotted as a function of membrane curvature and demonstrate that membrane curvature has a significant effect on the Atg8 conjugation reaction. Moreover, under comparable experimental conditions and Atg8 concentrations of 0.5 µM and below, we could not detect Atg8-PE formation on µm-sized GUVs but detected Atg8-PE formation with the SLiC-assay on nm-sized vesicles, compare Fig. 2A and 4A. Our data are supported by recent reports showing that Atg3 contains a curvature-sensing ALPS-motif [11], [26]. These results suggest that membrane curvature influences Atg8-PE formation *in vitro*. Curvature sensing of the conjugation reaction might present a mechanism for reducing the misallocation of Atg8-PE to endomembranes [27] which are characterized by a weakly curved membrane and for enhancing the targeting of the protein to curved membranes such as tubes, the rim of the phagophore and small vesicles. Further studies are needed to understand the influence of membrane curvature on Atg8-PE formation in detail.

## Conclusions

In this study, we have demonstrated that the ubiquitin-like protein Atg8 can actively induce and stabilize highly curved membrane structures such as nanotubes. The covalent anchorage of Atg8-PE to the membrane ensures the stability of such membrane shapes, since a decreasing Atg8 concentration in the aqueous solution does not induce a reduction of the surface density of membrane bound Atg8-PE. Our data suggest that the active membrane shaping by Atg8 is mediated via changes of the spontaneous curvature of the membrane during the covalent binding of Atg8 to the membrane lipids, by a curvature preference of the conjugation reaction and by the redistribution of the membrane bound protein according to local curvature. Consistent with in vivo data [8], such changes provide a direct control mechanism for the size and dynamics of various cellular organelles [7] and thus, will contribute to understanding their dynamics and biological function.

## Materials and Methods

### Materials

The phospholipids (DOPE, POPG, POPC, biotin-PEs) were purchased from Avanti Polar Lipids (Alabaster, AL). Cholesterol, BSA and Streptavidin were from Sigma-Adrich (St. Louis, MO). From Invitrogen (Carlsbad, CA), we obtained Texas Red-1,2-dihexadecanoyl-sn-glycero-3-phospho-ethanolamine (TR-DHPE) and amino-reactive AlexaFluor 488. Streptavidin conjugated microspheres with mean diameter of ∼6 µm were purchased from Polysciences, Inc. (Warrington, PA). Diluted lipid stock solutions at 4 mM were prepared with chloroform and stored in glass vials under Teflon seal at −20°C. The lipid mixture used for all vesicles contained 63.6 mol% DOPE, 13.6 mol% POPC, 13.6 mol% cholesterol and 9.1 mol% POPG. The proteins used in this study were purified as described previously [18]. Atg8 was labeled with Alexa 488 according to the protocol of the manufacturer. Free dye was removed by NAP-10 size exclusion columns and two dilution/concentration cycles with Vivaspin-20 (Sartorius). The functionality of the labeled Atg8 was checked by conjugation to PE and SDS-page [28] and was found to be unaffected by the label.

### Preparation of micropipettes

Micropipettes were pulled from glass capillaries (World Precision Instruments Inc., Sarasota, FL) using a pipette puller (Sutter Instruments, Novato, CA). Pipette tips were cut using a microforge (Narishige, Tokyo, Japan) at desired inner diameters of in the range 3–6 µm. Adhesion of the vesicles to the pipette was prevented by incubation of the pipette tips in 1 mg/mL fatty acid free BSA dissolved in 10 mM Tris-HCl pH 8.0 for about 5 min. Pipettes were filled from the back with 0.4 M sucrose solution.

### Giant Unilamellar Vesicles (GUVs)

The membrane lipid PE is essential for Atg8 conjugation to membranes. However, PEs with biologically relevant chain length (longer than 14 C-atoms) do not form stable lamellar phases at room temperature [29] and therefore do not form GUVs in the absence of other lipid components. Thus, only small fractions of PE (below 25 mol%) are typically employed for GUV formation. We did not detect Atg8-PE conjugation on GUVs with such a low DOPE concentration, which is in agreement with experiments on nm-sized vesicles [9], [10]. In vivo, typical concentrations of PE in membranes are 25-30 mol%. However, no reports on the phagophore membrane composition are known to us. In order to optimize Atg8 conjugation, we thus explored various compositions with higher PE fraction for which we could still from GUVs.

Cholesteryl hemisuccinate was reported to stabilize PE-membranes in a lamellar phase [30]. We chose PE fractions optimal for the Atg8 conjugation reaction: 60–70 mol% PE [9] supplemented with 15–20 mol% PC and 15–20 mol% cholesterol or cholesteryl hemisuccinate. In all cases we observed the formation of stable GUVs. In a following step we added minor fractions of anionic lipids to increase the rate of Atg8-PE formation [9], [10] and controlled the efficiency of the in-vitro-conjugation reaction with SDS-page [28]. The optimized lipid composition used throughout the paper contained 63.6 mol% DOPE, 13.6 mol% POPC, 13.6 mol% cholesterol and 9.1 mol% POPG. The GUVs exhibited homogeneous membranes, were unilamellar and stable at room temperature for several days. We observed significant Atg8 conjugation to such vesicles even in the absence of the Atg16-complex.

The lipid stock to grow GUVs was supplemented with 0.25 mol% Biotin-cap-DOPE and 0.5 mol% TR-DHPE. GUVs were prepared by electroformation [31] as described in detail in ref [32], [33]. Briefly, a few microliters of lipid solution were spread onto the conductive sides of two glass slides coated with indium tin oxide, The glasses were dried under vacuum for at least 2 h, assembled into a chamber using a 2 mm Teflon spacer. The chamber was filled with 0.4 M sucrose solution, closed with office clips and connected to an AC field generator (1.2 V, 10 Hz). After 2 h, the vesicle suspension was removed from the chamber and stored at room temperature until use. The vesicles were incubated with the Atgs in the same buffer as for the SLiC assay (50 mM Tris-HCl pH 8, 10 mM NaCl, 0.1 mM MgCl_2_) at a constant molar ratio Atg8: Atg7∶Atg3 5∶1∶1.

### Tube extraction and imaging

The observation chamber was assembled from two coverslips forming a sandwich with a 2 mm spacer. The chamber was open on both sides, which allowed the insertion of two horizontally aligned micropipettes. The chamber was filled with 3 µL 1 M NaCl, 0.2 µL of microsphere dispersion, 100 µl 0.4 M sucrose solution and ∼10 µl GUVs were added finally. The sample was let to rest for ∼10 minutes so that the beads and vesicles settled to the chamber bottom. The chamber was mounted on an inverted microscope (SP5, Leica Microsystems, Wetzlar, Germany) equipped with a 60×1.2 NA water immersion objective (Leica), 488 nm Argon laser and 594 nm NeHe laser. The detection wavelength windows for the labeled Atg8 and TR-DHPE were adjusted between 500–550 nm and in the range of 605–695 nm, respectively. All snapshots of GUVs shown in the paper are merged dual color images. The micropipettes were inserted into the chamber with the use of a three-dimensional micromanipulator system (Narishige, Japan). After insertion, zero pressure across the pipette tip was established by observing the flow of small fluorescent particles within the tip. The aspiration pressure was controlled through adjustments in the height of a connected water reservoir placed on µm-precise linear translational stages (Physik Instrumente, Karlsruhe, Germany). Vesicles with sufficient excess area and roughly 10 to 30 µm in diameter were selected. After prestressing of the GUV at 1 mN/m, a membrane-free bead was aspirated with a second pipette and positioned for about 1 minute in contact with the aspirated vesicle and then retracted slowly, forming a tube between the bead and the GUV. The lateral membrane tension σ was evaluated from the pipette aspiration pressure ΔP and the radii of the pipette, R_p_, and the GUV, R_GUV_, following the equation σ = ΔPR_p_/[2(1−R_p_/R_GUV_)] [34]. The membrane tension determines a corresponding tube radius. Intensities of isolated and adhering GUVs were measured from five averaged confocal scans. Because of the random orientation of the adhering couples in the scanning plane, the data were corrected for polarization effects. For the quantification of the intensities of the tube and the spherical part of the GUV, total intensity projections of the selected regions were obtained by ten non-averaged confocal z-scans within 3 µm height of the z-stack. In all cases, line plots with a width in the µm-range were fitted to a Gaussian function the integral of which yields the intensities of TR-DHPE, 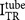 and 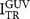 and the labeled Atg8, 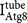 and 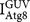, in the tube and the spherical GUV, respectively. The settings of the lasers and the detection photomultiplier were identical in all experiments. We took care that the illumination intensity was outside of the power range where photobleaching occurs [35] and generally reduced sample illumination as much as possible. The experiments were carried out at room temperature (21±2°C).

### Single liposome (SLiC) assay

The SLiC assay was performed as described in [36]. Shortly, the lipid film containing 0.5 mol% DSPE-Bio-PEG2000 and 0.5 mol% TR-DHPE was dried under vacuum at least 2 h, resuspended in 0.2 M sucrose and subjected to ten freeze thaw cycles. Such liposomes are unilamellar and characterized by a broad size distribution, see Fig. 4A, with a diameters larger than 20 nm [37]. Diluted liposomes (1 µM lipid) were immobilized on BSA-biotin coated glass surfaces via streptavidin coupling similar as described previously [37]. Freshly tethered liposomes were rinsed with buffer (50 mM Tris-HCl pH 8, 10 mM NaCl, 0.1 mM MgCl_2_) and incubated in the same buffer at room temperature. The protein concentrations used were 125 nM Atg8, 25 nM Atg7, 25 nM Atg3. The reaction was stopped after 10, 20 and 40 minutes by removing the protein solution and rinsing with buffer twice. The samples were imaged immediately by an inverted microscope (IX71; Olympus, Center Valley, PA) with a long-working distance water immersion objective (60×1.1 NA) with cover slip correction, and a back-illuminated electron multiplying charge-coupled device camera (ImageEM; Hamamatsu, Bridgewater, NJ). Images were evaluated applying a home written Matlab routine. It extracts single particle positions of background-corrected fluorescence images, identifies colocalized protein and liposome signals, and gives their intensities in both channels, 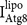 and 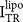, respectively. Since the fluorescence signal of nm-sized vesicles is proportional to their surface area, and the square root of the vesicle area is proportional to the vesicle size, we use 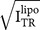 as a measure for the relative size of the vesicles. The liposome curvature is reciprocal of the radius. Note that Atg8-PE densities obtained with the SliC-assay are not comparable to those obtained from images of GUVs because of the different imaging conditions and equipment used.

## Supporting Information

S1 Video **Fluctuating tube.** 10 consecutive confocal scans illustrate strong shape fluctuations of the membrane tube, left: bead, right: GUV membrane. The relative time and the scale bar are shown. Both channels are merged. Image background and intensity are adjusted to visualize the tube.
(AVI)
